# *APC* Mutations Are Not Confined to Hotspot Regions in Early-Onset Colorectal Cancer

**DOI:** 10.3390/cancers12123829

**Published:** 2020-12-18

**Authors:** Alan Aitchison, Christopher Hakkaart, Robert C. Day, Helen R. Morrin, Frank A. Frizelle, Jacqueline I. Keenan

**Affiliations:** 1 Department of Surgery, University of Otago Christchurch, Christchurch 8011, New Zealand; frank.frizelle@cdhb.health.nz (F.A.F.); jacqui.keenan@otago.ac.nz (J.I.K.); 2 Mackenzie Cancer Research Group, Department of Pathology and Biomedical Science, University of Otago Christchurch, Christchurch 8011, New Zealand; christopher.hakkaart@otago.ac.nz; 3 Department of Biochemistry, University of Otago, Dunedin 9054, New Zealand; robert.day@otago.ac.nz; 4 Cancer Society Tissue Bank, University of Otago Christchurch, Christchurch 8011, New Zealand; helen.morrin@otago.ac.nz

**Keywords:** early-onset, colorectal cancer, APC, sequencing, DNA methylation, diet

## Abstract

**Simple Summary:**

Mutation of the *APC* gene is a common early event in colorectal cancer, however lower rates have been reported in younger cohorts of colorectal cancer patients. In sporadic cancer, mutations are typically clustered around a mutation cluster region, a narrowly defined hotspot within the *APC* gene. In this study we used a sequencing strategy aimed at identifying mutations more widely throughout the *APC* gene in patients aged 50 years or under. We found high rates of *APC* mutation in our young cohort that were similar to rates seen in older patients but the mutations we found were spread throughout the gene in a pattern more similar to that seen in inherited rather than sporadic mutations. Our study has implications both for the sequencing of the *APC* gene in early-onset colorectal cancer and for the etiology of this disease.

**Abstract:**

While overall colorectal cancer (CRC) cases have been declining worldwide there has been an increase in the incidence of the disease among patients under 50 years of age. Mutation of the *APC* gene is a common early event in CRC but is reported at lower rates in early-onset colorectal cancer (EOCRC) than in older patients. Here we investigate the *APC* mutation status of a cohort of EOCRC patients in New Zealand using a novel sequencing approach targeting regions of the gene encompassing the vast majority of known *APC* mutations. Using this strategy we find a higher rate (72%) of *APC* mutation than previously reported in EOCRC with mutations being spread throughout the gene rather than clustered in hotspots as seen with sporadic mutations in older patients. The rate of mutations falling within hotspots was similar to those previously seen in EOCRC and as such our study has implications for sequencing strategies for EOCRC patients. Overall there were low rates of both loss of heterozygosity and microsatellite instability whereas a relatively high rate (40%) of *APC* promoter methylation was found, possibly reflecting increasing exposure of young people to pro-oncogenic lifestyle factors.

## 1. Introduction

Over recent decades there has been an overall drop in the worldwide incidence rates of colorectal cancer (CRC) worldwide, primarily due to increased rates of colonoscopy screening which leads to the early detection and removal of precancerous polyps [1,2]. However, over the same period there has been a worldwide increase in incidence of early-onset colorectal cancer (EOCRC) [3,4]. While hereditary syndromes such as Lynch Syndrome [5] and familial adenomatous polyposis (FAP) [6] account for a small proportion of cases [7], and an even smaller proportion arise due to long-standing inflammatory bowel disease [8], the majority of these cancers are considered to be sporadic [9]. Whereas the majority of EOCRC patients are not found to have an inherited disorder, they are more likely to have a family history of disease compared to older patients [10]. Against this complex background, the underlying causative factors that initiate the genetic mutations required to drive the adenoma-carcinoma sequence in these individuals are not well understood.

Mutation of the tumour-suppressor adenomatous polyposis coli (*APC)* gene is the most frequent early genetic event in the progression of colorectal carcinogenesis. *APC* mutations are found in the earliest microscopic adenomas [11], and at the same rate in early adenomas as in sporadic tumours [12]. APC is a multi-functional protein in epithelial cells, having roles in Wnt signalling [13], cell structure [14], epithelial cell polarity [15], and apoptosis [16]. Consequently, the loss of a fully functioning APC protein can result in cells that display a number of pro-carcinogenic characteristics.

Both alleles of *APC* require inactivation for carcinogenesis to occur [11]. This may involve mutation of both alleles, or the second allele may be silenced by loss of heterozygosity (LOH) [17]. However, the worldwide increase in early onset CRC hints more at the involvement of environmental and/or lifestyle factors [1], and this is reinforced by studies on people who migrate from low- to high-risk areas of the world [18]. Accordingly, epigenetic modification of the promoter region of this gene can also result in altered APC expression in the absence of mutated DNA sequence, as recently reported in a mouse model of obesity [19].

Despite mutations of the *APC* gene being well documented in colorectal cancers as a whole, the incidence of mutations is reported at notably lower rates in EOCRC than in older patients [20,21], adding to speculation that sporadic EOCRC may be a subtype of CRC with distinct clinical and molecular features [10,22,23] that reflect alternative pathways for the development of CRC. For example, 15 to 30% of CRC cases develop from serrated polyps [24], a subset of CRCs that rarely present with *APC* mutations but commonly have mutations in *BRAF* and evidence of microsatellite instability [25]. However, another possibility may also exist. The *APC* gene is located on chromosome 5q21-q22 and consists of 15 exons, with the final exon making up more than 75% of the coding sequence of the gene. It is this final exon where the bulk of mutations reportedly occur, specifically within a mutation cluster region (MCR) between codons 1286 and 1513 [26]. Consequently, it is this hotspot where many studies investigating *APC* mutation have focused their efforts [27,28,29,30], raising the possibility those *APC* mutations that fall outside this region are not detected.

The aim of this study was to explore the molecular genotype of a cohort of tumour tissues collected from EOCRC patients in Canterbury, New Zealand. The first objective was to identify whether the rate of *APC* mutation in this cohort was similar to that reported elsewhere [20,21]. For this study however a sequencing approach was developed to maximise coverage of the *APC* gene, enabling identification of both the presence and location of mutations throughout the *APC* gene as a whole. Further investigation was undertaken to determine whether loss of heterozygosity [17] and/or methylation of the *APC* gene promoter region [31] may have contributed to silencing of the second *APC* gene allele in these early-onset colorectal cancers. The occurrence, type, and location of *APC* mutations are reported, as is evidence of microsatellite instability, loss of heterozygosity, and *APC* promoter methylation, and these findings are compared to clinical features of the tumours.

## 2. Results

The cohort of 25 early-onset CRC cases consisted of 14 (56%) women and 11 (44%) men aged 50 years or under at diagnosis (median 44 years, range 28–50 years) (Table 1). The tumours were predominantly distal (17/25, 68%). All but two tumours were classed as adenocarcinomas (92%), with a single adenosquamous carcinoma (4%) and a single signet-ring cell carcinoma (4%) completing the cohort (Table 1).

Twenty four mutations meeting the inclusion criteria for absolute (>10) and relative (>20%) read number were found in 18/25 (72%) tumour samples and confirmed by Sanger sequencing. Twelve tumours had a single *APC* mutation while six tumours had two mutations. The mutations consisted of 14 nonsense, six frameshift, three missense, and one splice region mutation (Table 2). Sequencing of matched normal tissues revealed that while the majority of mutations were sporadic, two *APC* mutations were determined to be germline as they were also present in matched normal tissue.

One of the germline mutations (a *c.3374T>C* variant) results in a substitution at codon 1125 of a valine to an alanine (p.V1125A) and is regarded as a conservative change as they are closely related amino acids. Coupled with the occurrence of the variant in the general population [41], as well as its position in a poorly conserved region of the gene, the *c.3374T>C* variant is considered benign.

**Figure 1:**
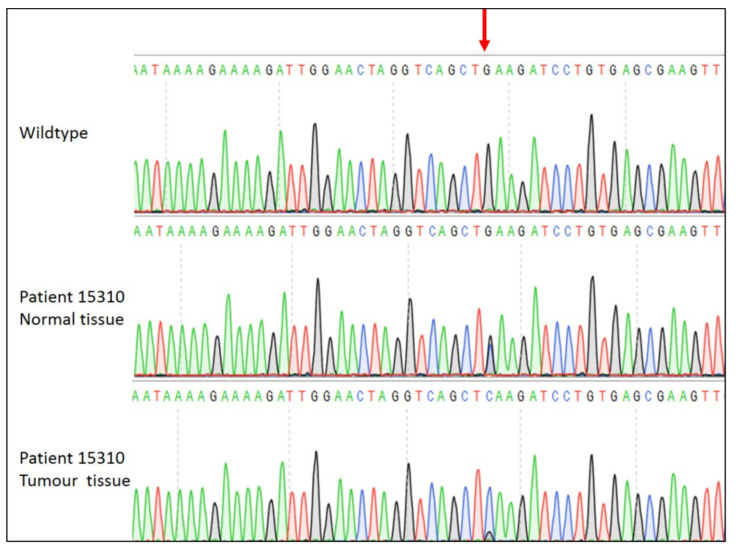
Sanger sequencing of *APC c.3949G>C* mutation. The guanine base indicated by the red arrow in the wild type *APC* sequence is mutated to a cytosine in one allele in the normal tissue of the patient to give a heterozygous sequence. In the tumour tissue the wild type guanine allele is barely visible.

However, the second germline mutation, *c.3949G>C*, which results in a glutamic acid to glutamine change at codon 1317 of APC (p.E1317Q), was accompanied by loss of the wild type allele in the tumour tissue (Figure 1). This mutation was found in a 36 year old female (Patient 15310) with a stage 2 adenocarcinoma with no lymph node involvement or metastasis. Subsequent colonoscopies have shown the patient to be disease-free up to eight years post-surgery with no evidence of polyps despite the germline *APC* mutation. There was no reported family history of bowel cancer.

## 3. Distribution of APC Mutations

**Figure 2:**
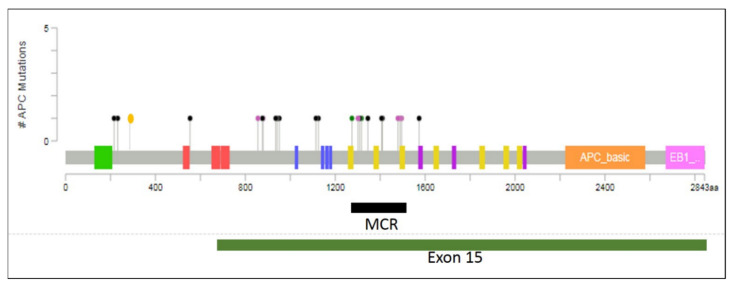
*APC* mutations found in EOCRC samples mapped to their position within the protein. Each lollipop represents the position of a mutation with the colours of the lollipop representing different types of mutation: black, nonsense mutations; green, missense mutation; pink, frameshift mutation; yellow, splice variant. While a high proportion of mutations occur within the mutation cluster region (MCR, black bar), mutations are found throughout the first 1600 codons of *APC* including some upstream of exon 15 (green bar). Protein domains: green, oligomerisation; red, armadillo repeats; blue, 15 amino acid repeats; yellow, 20 amino acid repeats; purple, SAMP motif. The plot was generated using Mutation Mapper (www.cbioportal.org) and manually curated.

The mutations found in the early-onset cohort were distributed widely across the first 1600 codons of *APC*. While only 8/25 (32%) of patients had mutations located within the mutation cluster region [26], a total of 14/25 (56%) of patients had one or two mutations in the large final exon of APC and that figure rose to 18/25 (72%) throughout the whole gene (Figure 2).

## 4. Microsatellite Instability

Of the 24 samples that were stained for mismatch repair proteins, 21 stained for all four mismatch repair proteins and were therefore considered microsatellite stable. All three of the remaining samples displayed microsatellite instability, with one negative for MSH6 staining and two negative for PMS2 staining. The three mismatch repair deficient tumours did not cluster at any location in the colon, as they were found in the ascending colon, splenic flexure and the sigmoid colon, respectively. Neither was there any association of microsatellite instability with age or gender.

One of the 21 samples that stained positively for all four mismatch repair genes was reported to have a *BRAF* mutation. This sample was the only adenosquamous carcinoma in the cohort. This combination of a *BRAF* mutation in a microsatellite stable adenosquamous colon cancer has been reported before [42].

## 5. Loss of Heterozygosity is Associated with *APC* Mutation Near Codon 1300

Loss of heterozygosity at the *APC* locus was found in 3/25 (12%) samples in the cohort. Strikingly, the three samples with LOH all carried an *APC* mutation clustered around codon 1300, a phenomenon previously reported in sporadic CRC [43]. As with microsatellite stability, no association was seen between LOH and any of age, gender or tumour location.

## 6. Methylation of the *APC* Promoter

Methylation analysis of the CpG island at the *APC* 1A promoter, responsible for the predominant APC isoform, was detected in 10/25 (40%) of tumours, a higher rate than previously reported in CRC [31], although there has been at least one recent report of higher *APC* promoter methylation in CRC [44]. There was no association of *APC* promoter methylation with age (mean age 43.5 years vs 44 years for the whole cohort), gender (60% vs. 56% female) or tumour location (70% vs. 68% distal).

## 7. Discussion

In this study we found a higher than anticipated rate of *APC* gene mutation in a cohort of EOCRC tumours, with sporadic mutations occurring throughout the gene rather than clustering in one region as expected. The *APC* gene is long, encompassing 8529 bases of coding sequence over multiple exons covering over 100 kilobases of chromosome 5, making targeted sequencing strategies cumbersome and expensive. Therefore, previous studies have focused on mutation hotspots in the gene, particularly the mutation cluster region between amino acids 1286 and 1513 [26] or more widely within the final, large exon which encompasses the majority of *APC* mutations [45]. Although targeted sequencing is seen as cost-effective, it has the potential to miss mutations that fall outside these regions. Accordingly, we developed a sequencing strategy to maximize the mutations identified while maintaining a manageable amount of sequencing.

Regions of *APC* with very low rates of mutation in sporadic CRCs were identified using the Catalogue of Somatic Mutation In Cancer (COSMIC) database, and excluded from our sequencing strategy. In this study, exons 5, 6, 8, 9, 11, 12 and 13, as well as exon 15 from amino acids 788 to 1593 were targeted. These exons encompass the vast majority of non-synonymous mutations of the *APC* gene in the COSMIC database, and each was sequenced. This approach identified 24 mutations in 18/25 (72%) tumour samples across these regions of the *APC* gene, a notably higher rate than some previous studies looking at *APC* mutation in early-onset CRCs [20,21] that may, in part, reflect the extent of *APC* gene coverage by the methodology used here. Importantly, if we had only sequenced the mutation cluster region, this number would have dropped to eight out of 25 tumours (32%). Likewise, mutations in the final exon of the gene were identified in only 14/25 (56%) of the samples. These findings convincingly highlight that protocols applied to study *APC* mutations are of critical importance, and that studies limited to “hotspot” regions of the gene may significantly under-report the mutation rate.

Despite known familial colorectal cancer syndromes being excluded, two patients were found to have germline *APC* mutations. While the p.V1125A variant was deemed to be benign, the p.E1317Q variant has been associated with colorectal cancer previously [39]. Notably, p.E1317Q is a missense mutation, resulting in a glutamic acid to glutamine change rather than the more common truncated APC proteins that result from *APC* mutations in FAP [46]. While the matched normal tissue showed both mutant and wild type alleles, the tumour tissue showed only mutant allele present (Figure 1) and this sample was one of three to display LOH at the *APC* locus. Accordingly, the lack of family history might be due to the single mutant allele alone not being sufficient for carcinogenesis, suggesting a second genetic hit is required for the pathogenicity of the p.E1317Q mutation to occur, an example of the damaging potential caused by loss of heterozygosity. It was noted that this patient did not present with polyps and would therefore be unlikely to be considered as a possible case of FAP and therefore not tested for *APC* mutation. As such, although we cannot rule out the possibility that the mutation has arisen de novo in this patient, it is likely that this patient has an atypical FAP phenotype and our finding may have implications for FAP testing.

We found that 8/24 (33%) mutations occurred within the MCR as defined by Miyoshi and colleagues [26]. Removing the two germline mutations made little difference to the proportion of sporadic mutations in the MCR (7/22, 32%). This is lower than might have been expected based on previous studies of CRC [45] where the majority of somatic mutations occur within the MCR.

Mutations in *APC* predominantly result in truncated proteins that retain some function essential for the survival of the cells [47]. The location of these truncating *APC* mutations affects the properties of the resultant protein and the tumour phenotype [48]. While familial *APC* mutations are scattered across the 5′ half of the gene, with notable hotspots at codons 1061 and 1309, somatic mutations tend to occur within the MCR [45]. This region contains a number of 20-amino acid repeats that act as β-catenin binding sites. The location of a mutation within the MCR determines how many of these repeats are included in the truncated protein.

Kohler and colleagues showed that mutations in the MCR resulted in the disruption of the third 20-amino acid repeat region while maintaining the first 20aa repeat. They also showed by co-immunoprecipitation that the second 20 amino acid repeat region, unlike the other two, had no β-catenin binding capacity therefore all the mutations within the MCR produced truncated proteins with similar β-catenin binding efficiency [49]. Our finding of an increased proportion of *APC* mutations occurring outside the MCR in EOCRC suggests that the β-catenin binding capacity of truncated APC is of less importance in EOCRC than in older patients, and that alternative functions of APC may be at play in these tumours.

Three decades ago biological differences were identified in tumours located in either the proximal (right-sided) or distal (left-sided) colon, leading to the proposal of distinct categories of colorectal cancer based on tumour location [50]. In particular, proximal tumours had characteristics similar to Lynch Syndrome, while distal tumours displayed characteristics more familiar to FAP. Moreover, EOCRC patients present more often with distal tumours than older CRC patients. One study had 32% of patients aged 35–39 diagnosed with having tumours in their rectum, with the percentage in subsequent age groups decreasing, down to 1% in those aged over 85 years. Conversely, only 9.3% of the 35–39 years age group had tumours in the caecum, rising to 23.2% in the over 85 year olds [51]. Further to Bufill’s categorisation of CRC by tumour location, it has been suggested that EOCRC should be similarly categorised by location [52].

This leads to an intriguing hypothesis. *APC* mutations in the MCR that result in 2–3 intact 20-amino acid repeats are more likely to be found in proximal tumours, while mutations leaving only 1 or no 20-amino acid repeats tend to be present in distal CRC [48]. Inherited mutations in FAP occur more widely across the 5′ half of the *APC* gene than somatic mutations, of which a large proportion are found in the MCR [45]. Our finding that *APC* mutations in EOCRC more closely resembled the pattern of *APC* mutations seen in FAP fits with the observation that EOCRC tumours are predominantly distal. In support of this hypothesis is the observation that the majority of EOCRC tumours do not show microsatellite instability and those that do are usually due to Lynch Syndrome [53]. In population-based studies MSI is found in between 7% and 17% of CRC cases under the age of 50 years [54]. Our finding of MSI in three out of 24 (12.5%) tumours for which MMR staining was available fits with these previous findings.

The CpG island methylator phenotype (CIMP), where multiple tumour suppressor genes are silenced by promoter methylation, is reportedly found less in EOCRC patients than in older-onset CRC patients, with one study reporting the CIMP to be absent in all 47 EOCRCs, but present in 15/49 (31%) of CRCs in over 60 year olds [10]. However, we found promoter methylation of the *APC* gene in 40% of EOCRC tumours, a higher rate than reported in overall CRC [31], suggesting that specific methylation at the *APC* gene promoter may play an important role in EOCRC, despite lower methylation more generally. A previous study of patients presenting with CRC or colorectal polyps found *APC* methylation present in 8/50 (16%) samples [55]. When this cohort was stratified by age however the rate increased to 6/11 samples (55%) in those patients under 60 years old, reinforcing the idea that *APC* promoter methylation may play a significant role in EOCRC.

While diet is a major environmental factor proposed to influence DNA methylation in the colon [56], a finding of promoter methylation of the *APC* gene does not inform a direct role for diet in the genesis of early onset CRC. Moreover, whether this effect is the result of early maternal nutrition that can markedly affect the epigenetic patterning in the fetus is also beyond the scope of this study. However, our finding of tumour-specific *APC* promoter methylation in 40% early-onset CRC, with or without retention of a homozygous *APC* gene, highlights growing awareness that environmental factors need to be taken into consideration as we try to find ways to reduce the number of young people developing this disease.

## 8. Methods

### 8.1. Cohort

Samples from 25 patients with early-onset colorectal cancer, diagnosed at or under the age of 50 years, were obtained from the Cancer Society Tissue Bank, Christchurch, New Zealand. Patients with known hereditary syndromes, HNPCC or FAP were excluded. The samples consisted of frozen tumour tissue as well as matched normal tissue taken at the time of surgery. The study was granted ethics approval by the University of Otago Human Ethics Committee (H18/143).

### 8.2. DNA Extraction

DNA was extracted from up to 25 mg of frozen tissue using a DNeasy Blood and Tissue kit (Qiagen, Hilden, Germany). Prior to extraction the tissue was homogenized in a Precellys Evolution homogenizer (Bertin Technologies, Montigny-le-Bretonneux, France) with 180 µL lysis buffer using 2.8 mm zirconium oxide beads in reinforced 2 mL microtubes. DNA was then extracted according to the manufacturer’s instructions.

### 8.3. Sequencing Library Preparation

The *APC* gene sequence of the tumour samples and matched normal tissue was determined by running pooled, indexed PCR products on an Illumina MiSeq instrument following the previously described protocol [57]. Briefly, dual-indexed amplicon sequencing libraries for *APC* were generated using a two-step PCR approach. In the first PCR step, regions of the *APC* gene, accounting for the vast majority of known cancer-associated mutations, were amplified. *APC*-specific primers were designed with an additional 18 bp of known non-specific sequence that was used as a priming site for the second PCR reaction (Table S1). PCR products from the same study participant and sample were pooled and purified using HighPrep beads (MagBio, Gaithersburg, MD, USA). In the second PCR step, pooled PCR products were amplified using a unique pair of indexed primers designed to add sequences necessary for multiplex sequencing on an Illumina MiSeq (Illumina, San Diego, CA, USA) (Tables S2 and S3).

To prepare the amplicon library for sequencing, sample-specific libraries were pooled, purified using HighPrep beads, and quantified with the Qubit dsDNA HS Assay Kit (Thermo Fisher (Waltham, MA, USA). Sequencing libraries were run on a DNA7500 Bioanalyzer chip (Illumina, San Diego, CA, USA) to determine the average library size. Libraries were sequenced on an Illumina MiSeq using V2-500 cycle reagent kits (Illumina, San Diego, CA, USA).

### 8.4. Sequence Analysis and Annotation

Raw paired end reads were cleaned with Trimmomatic v.0.35 [58]. Cleaned reads were aligned to the human reference genome (GRCh37/hg19) using the Burrows-Wheeler Aligner v.0.7.10 [59]. Amplicons were sequenced to a minimum depth of 40 reads. Single nucleotide variants and insertion/deletion variants were called using ‘The Genome Analysis Toolkit’ (GATK) v.3.6 [60]. The effects of variants were predicted using SnpEff v.4.2 [61]. All putative mutations were visually inspected using the Integrative Genomics Viewer [62]. The functional consequences of missense variants were predicted in silico using five different models that included SIFT [63], Provean [64], Mutation Assessor [65], and PolyPhen2 (using both HumDiv and HumVar datasets) [66]. Mutations predicted to be deleterious by at least 3/5 predictive models were considered of interest. Mutations with low absolute (<10) or relative (<20%) allele counts were excluded. Mutations discovered by this method were confirmed by Sanger sequencing.

### 8.5. LOH at APC Locus

The *APC* sequences obtained above were used to detect loss of heterozygosity (LOH) by comparing levels of each allele at four single nucleotide polymorphisms (SNPs) within the *APC* gene. SNPs showing heterozygosity, by presence of both alleles, in normal tissue were considered to have LOH if the read count of one allele was reduced in relation to the alternate allele in tumour tissue. A low number of alleles present for the missing allele likely reflected non-tumour cells, such as lymphocytes, within the tumour tissue. The four SNPs analysed were rs2229992, rs351771, rs1801166 and rs41115. If all the informative SNPs within the *APC* gene in a single patient showed loss of an allele in the tumour tissue then the tumour was considered to display LOH at the *APC* locus.

#### Sanger Sequencing

Mutations detected using the method above were confirmed by Sanger sequencing. Briefly, purified PCR products were prepared for sequencing using BIG Dye chemistry (Applied Biosystems, Foster City, CA, USA) and sequenced on an ABI3700xl Genetic Analyser (Applied Biosystems, Foster City, CA, USA).

### 8.6. Microsatellite Instability

Slides of sectioned tumour tissue were available from 24 of the 25 patients, and these were stained for mismatch repair proteins to assess microsatellite instability [67]. Briefly, following incubation with antigen retrieval solution, consecutive slides were immunostained with primary antibodies to the mismatch repair proteins MSH2, MSH6, MLH1, and PMS2. Slides were then incubated with DAB to visualise primary antibody staining, followed by counterstaining with haematoxylin and finally bluing agent. All slides were scored by a clinical pathologist.

### 8.7. Bisulphite Treatment and Methylation Analysis

DNA was modified by sodium bisulphite using EZ DNA Methylation-Gold kit (Zymoresearch, Irvine, CA, USA) according to the manufacturer’s protocol. Methylation-specific PCR (MSP) was used to amplify methylated and unmethylated *APC* promoter 1A sequences using previously published conditions and primers [31].

## 9. Conclusions

Our study of a cohort of colorectal tumours from patients diagnosed under the age of 50 years identified *APC* mutations at a rate similar to that found in later-onset CRC, suggesting that similar mechanisms underlie tumour development in both age groups and that the earlier onset seen in the current cohort may be due to environmental factors. However, we found that the distribution of *APC* mutations more closely resembled that of familial *APC* mutations than sporadic later-onset CRC, despite the mutations being almost exclusively sporadic. This has implications for *APC* sequencing techniques as methodologies focusing on the mutation cluster region rather than the whole gene may miss a substantial number of *APC* mutations in sporadic EOCRC.

## Figures and Tables

**Table 1:** Clinical features of the early-onset CRC cohort.

	Number	%
**Total**	25	
**Gender**		
Male	11	44
Female	14	56
**Age at diagnosis (years)**		
<30	1	4
31–40	6	24
41–50	18	72
**Tumour site**		
Caecum	1	4
Ascending colon	6	24
Hepatic flexure	1	4
Splenic flexure	1	4
Descending colon	3	12
Sigmoid colon	12	48
Rectosigmoid	1	4
**Pathology**		
Adenocarcinoma	23	92
Adenosquamous carcinoma	1	4
Signet-ring cell carcinoma	1	4

**Table 2:** Molecular characteristics of EOCRC patients.

Patient Number	Microsatellite Instability	Mutation			Mutational Outcome	Mutational Assessment ^1^	APC Methylation	LOH	Reference
		DNA	rsID	Protein					
10006	MSI	*c.2626C>T*	rs121913333	p.R876X	Nonsense	Damaging	U	-	[32]
10765	MSS	*c.835-8A>G*	rs1064793022	Splice variant	Truncation ^2^	Damaging	U	-	[33]
12294	MSS	*c.1660C>T*	rs137854573	p.R554X	Nonsense	Damaging	M	-	[34]
12961	MSS	*c.2853T>G*	rs575406600	p.Y951X	Nonsense	Damaging	M	-	[34]
13046	MSS	*c.4488delT*	n/a	p.P1497fsX	Frameshift	Damaging	U	-	This Study
		*c.2805C>A*	rs137854575	p.Y935X	Nonsense	Damaging			[35]
13501	MSS	*c.2636delA*	n/a	p.Q879fsX	Frameshift	Damaging	U	-	This Study
13564	MSS	*c.4033G>T*	rs1211642532	P.E1345X	Nonsense	Damaging	U	LOH	[36]
14459	MSS	*c.4463delT*	rs1114167577	p.L1488fsX	Frameshift	Damaging	M	-	[37]
		*c.3826delT*	n/a	p.S1276fsX	Frameshift	Damaging			This Study
15052	MSS	*-*	-	-	-	-	M	-	-
15296	MSS	*-*	-	-	-	-	U	-	-
15310	MSS	*c.4216C>T*	rs587782518	p.Q1406X	Nonsense	Damaging	U	LOH	[38]
		***c.3949G>C***	**rs1801166**	**p.E1317Q**	**Missense**	**Benign**			[39]
15471	MSS	*-*	-	-	-	-	M	-	-
16872	MSS	*-*	-	-	-	-	U	-	-
16993	MSS	*c.4717G>T*	rs878853217	p.E1573X	Nonsense	Damaging	U	-	[40]
		*c.2821G>T*	n/a	p.E941X	Nonsense	Damaging			[37]
17068	MSS	*-*	-	-	-	-	M	-	-
17871	MSS	*c.4485delT*	n/a	p.S1459fsX	Frameshift	Damaging	U	-	This Study
		*c.2626C>T*	rs121913333	p.R876X	Nonsense	Damaging			[32]
18090	MSS	*c.3925G>T*	n/a	p.E1309X	Nonsense	Damaging	M	LOH	[27]
19199	MSS	*c.646C>T*	rs62619935	p.R216X	Nonsense	Damaging	U	-	[36]
19513	MSS	*c.3740C>T*	n/a	p.A1247V	Missense	Benign	M	-	This Study
		*c.3340C>T*	rs121913331	p.R1114X	Nonsense	Damaging			[40]
19906	MSS	*-*	-	-	-	-	U	-	-
20039	MSS	*c.4230C>A*	n/a	p.C1410X	Nonsense	Damaging	U	-	This Study
20085	MSI	*c.694C>T*	rs397515734	p.R232X	Nonsense	Damaging	M	-	[35]
20187	MSS	***c.3374T>C***	**rs377278397**	**p.V1125A**	**Missense**	**Benign**	U	-	[41]
21025	MSI	*c.2563_2564dupGA*	n/a	p.R856fsX	Frameshift	Damaging	M	-	This Study
21082	MSS	*-*	-	-	-	-	U	-	-

^1^ Missense mutations were considered damaging if 3/5 or more software tools predicted the mutation to be possibly or probably damaging. Nonsense or frameshift mutations were all considered damaging without predictive tools. ^2^ The splice variant *c.835-8A>G* results in a frameshift that ultimately leads to premature truncation of *APC*. Mutations in **bold** denote germline status. MSS, microsatellite stable; MSI, microsatellite instability; M, methylated APC promoter; U, Unmethylated APC promoter; LOH, Loss of heterozygosity at the *APC* locus; n/a, these mutations have no rsID.

## Supplementary Materials

The following are available online at https://www.mdpi.com/2072-6694/12/12/3829/s1, Table S1: APC amplicon specific primers for next generation sequencing, Table S2: Forward adapter primers for creating next generation sequencing libraries, Table S3: Reverse adapter primers for creating next generation sequencing libraries.
