# Supplementary material for: APC Mutations Are Not Confined to Hotspot Regions in Early-Onset Colorectal Cancer
Source: Cancers (Basel). 2020 Dec 18;12(12):3829. doi: 10.3390/cancers12123829 (PMC7766084; doi:10.3390/cancers12123829)
Supplement: Supplementary file 1 [file cancers-12-03829-s001.pdf]

Supplementary Materials: *APC* Mutations Are Not Confined to Hotspot Regions in Early-Onset Colorectal Cancer

Alan Aitchison, Christopher Hakkaart, Robert C. Day, Helen R. Morrin, Frank A. Frizelle and Jacqueline I. Keenan

Table S1. ***APC*** **amplicon specific primers for next generation sequencing**

Name	Sequence (5′-3′)	Product Size (bp)
APC Exon 5 F	ACGACGCTCTTCCGATCTCACCATGACTGACGTATTTGCT	389
APC Exon 5 R	CGTGTGCTCTTCCGATCTAGAGCCAAAATAAACACAGCCTT	
APC Exon 6 F	ACGACGCTCTTCCGATCTCCTGAGCTTTTAAGTGGTAGCC	458
APC Exon 6 R	CGTGTGCTCTTCCGATCTTGTAAACTGACAGCTAAAGTAAGGT	
APC Exon 8 F	ACGACGCTCTTCCGATCTTCTGCAGTTTAATGCTCATATGCAA	402
APC Exon 8 R	CGTGTGCTCTTCCGATCTTGGCATTAGTGACCAGGGTTT	
APC Exon 9 F1	ACGACGCTCTTCCGATCTTCATCACTTAATTGGTTTTTGGCTT	397
APC Exon 9 R1	CGTGTGCTCTTCCGATCTAAGGACTCGGATTTCACGCC	
APC Exon 9 F2	ACGACGCTCTTCCGATCTGGAAATTCCCGGGGCAGTAA	374
APC Exon 9 R2	CGTGTGCTCTTCCGATCTTGAGTAGCACAAATGGCTGA	
APC Exon 11 F1	ACGACGCTCTTCCGATCTGGGGTGGAGAAACTGGCATA	406
APC Exon 11 R1	CGTGTGCTCTTCCGATCTACCTTGTTGGCTACATCTCCAAA	
APC Exon 11 F2	ACGACGCTCTTCCGATCTTAGGGGGACTACAGGCCATT	320
APC Exon 11 R2	CGTGTGCTCTTCCGATCTGCGAATGTGAAGCACAGGTT	
APC Exon 12 F	ACGACGCTCTTCCGATCTTGGCTTCAAGTTGTCTTTTTAATG	511
APC Exon 12 R	CGTGTGCTCTTCCGATCTTGAGATACTAAATACTGAGCAACAA	
APC Exon 13 F	ACGACGCTCTTCCGATCTAGTGATAGGATTACAGGCGTG	476
APC Exon 13 R	CGTGTGCTCTTCCGATCTGAAATTAGGGAATCTCATGG	
APC Exon 15 F1	ACGACGCTCTTCCGATCTTCCCAAGGCATCTCATCGT	387
APC Exon 15 R1	CGTGTGCTCTTCCGATCTATGGCTGACACTTCTTCCATGA	
APC Exon 15 F2	ACGACGCTCTTCCGATCTAGAAGCTCTGCTGCCCATAC	387
APC Exon 15 R2	CGTGTGCTCTTCCGATCTCCTTCCAGAGTTCAACTGCTCA	
APC Exon 15 F3	ACGACGCTCTTCCGATCTTCCATACAGGTCACGGGGAG	436
APC Exon 15 R3	CGTGTGCTCTTCCGATCTTGGATGGAGCTGATTCTGCC	
APC Exon 15 F4	ACGACGCTCTTCCGATCTAGCAGTGAGAATACGTCCACA	449
APC Exon 15 R4	CGTGTGCTCTTCCGATCTGCTTTGTGCCTGGCTGATTC	
APC Exon 15 F5	ACGACGCTCTTCCGATCTGCAGACTGCAGGGTTCTAGT	470
APC Exon 15 R5	CGTGTGCTCTTCCGATCTGACCCTCTGAACTGCAGCAT	
APC Exon 15 F6	ACGACGCTCTTCCGATCTAACAGCTCAAACCAAGCGAG	504
APC Exon 15 R6	CGTGTGCTCTTCCGATCTTTTGAAGCAGTCTGGGCTGG	

Amplicon-specific primers (black bases) designed with non-specific 18bp sequences at the 5′ end of primers (red bases). Two non-specific sequences used, one for forward primers, one for reverse primers. bp, base pairs.

Table S2. Forward adapter primers for creating next generation sequencing libraries

Name	Sequence (5'-3')
FPI1	AAT GAT ACG GCG ACC ACC GAG ATC TAC AC **CGTGAT** ACAC T CTT TCC CTA CAC GAC GCT CTT CCG ATC T
FPI2	AAT GAT ACG GCG ACC ACC GAG ATC TAC AC **ACATCG** ACAC T CTT TCC CTA CAC GAC GCT CTT CCG ATC T
FPI3	AAT GAT ACG GCG ACC ACC GAG ATC TAC AC **GCCTAA** ACAC T CTT TCC CTA CAC GAC GCT CTT CCG ATC T
FPI4	AAT GAT ACG GCG ACC ACC GAG ATC TAC AC **TGGTCA** ACAC T CTT TCC CTA CAC GAC GCT CTT CCG ATC T
FPI5	AAT GAT ACG GCG ACC ACC GAG ATC TAC AC **CACTGT** ACAC T CTT TCC CTA CAC GAC GCT CTT CCG ATC T
FPI6	AAT GAT ACG GCG ACC ACC GAG ATC TAC AC **ATTGGC** ACAC T CTT TCC CTA CAC GAC GCT CTT CCG ATC T
FPI7	AAT GAT ACG GCG ACC ACC GAG ATC TAC AC **GATCTG** ACAC T CTT TCC CTA CAC GAC GCT CTT CCG ATC T
FPI8	AAT GAT ACG GCG ACC ACC GAG ATC TAC AC **TCAAGT** ACAC T CTT TCC CTA CAC GAC GCT CTT CCG ATC T
FPI9	AAT GAT ACG GCG ACC ACC GAG ATC TAC AC **CTGATC** ACAC T CTT TCC CTA CAC GAC GCT CTT CCG ATC T
FPI10	AAT GAT ACG GCG ACC ACC GAG ATC TAC AC **AAGCTA** ACAC T CTT TCC CTA CAC GAC GCT CTT CCG ATC T
FPI11	AAT GAT ACG GCG ACC ACC GAG ATC TAC AC **GTAGCC** ACAC T CTT TCC CTA CAC GAC GCT CTT CCG ATC T
FPI12	AAT GAT ACG GCG ACC ACC GAG ATC TAC AC **TACAAG** ACAC T CTT TCC CTA CAC GAC GCT CTT CCG ATC T
FPI13	AAT GAT ACG GCG ACC ACC GAG ATC TAC AC **TTGACT** ACAC T CTT TCC CTA CAC GAC GCT CTT CCG ATC T
FPI14	AAT GAT ACG GCG ACC ACC GAG ATC TAC AC **GGAACT** ACAC T CTT TCC CTA CAC GAC GCT CTT CCG ATC T
FPI15	AAT GAT ACG GCG ACC ACC GAG ATC TAC AC **TGACAT** ACAC T CTT TCC CTA CAC GAC GCT CTT CCG ATC T
FPI16	AAT GAT ACG GCG ACC ACC GAG ATC TAC AC **GGACGG** ACAC T CTT TCC CTA CAC GAC GCT CTT CCG ATC T

Forward adapter primers with unique index sequences shown in **red**.

Table S3. Reverse adapter primers for creating next generation sequencing libraries

Name	Sequence (5'-3')
RPI1	CAA GCA GAA GAC GGC ATA CGA GAT **CGTGAT** GTG ACT GGA GTT CAG ACG TGT GCT CTT CCG ATC
RPI2	CAA GCA GAA GAC GGC ATA CGA GAT **ACATCG** GTG ACT GGA GTT CAG ACG TGT GCT CTT CCG ATC
RPI3	CAA GCA GAA GAC GGC ATA CGA GAT **GCCTAA** GTG ACT GGA GTT CAG ACG TGT GCT CTT CCG ATC
RPI4	CAA GCA GAA GAC GGC ATA CGA GAT **TGGTCA** GTG ACT GGA GTT CAG ACG TGT GCT CTT CCG ATC
RPI5	CAA GCA GAA GAC GGC ATA CGA GAT **CACTGT** GTG ACT GGA GTT CAG ACG TGT GCT CTT CCG ATC
RPI6	CAA GCA GAA GAC GGC ATA CGA GAT **ATTGGC** GTG ACT GGA GTT CAG ACG TGT GCT CTT CCG ATC
RPI7	CAA GCA GAA GAC GGC ATA CGA GAT **GATCTG** GTG ACT GGA GTT CAG ACG TGT GCT CTT CCG ATC
RPI8	CAA GCA GAA GAC GGC ATA CGA GAT **TCAAGT** GTG ACT GGA GTT CAG ACG TGT GCT CTT CCG ATC
RPI9	CAA GCA GAA GAC GGC ATA CGA GAT **CTGATC** GTG ACT GGA GTT CAG ACG TGT GCT CTT CCG ATC
RPI10	CAA GCA GAA GAC GGC ATA CGA GAT **AAGCTA** GTG ACT GGA GTT CAG ACG TGT GCT CTT CCG ATC
RPI11	CAA GCA GAA GAC GGC ATA CGA GAT **GTAGCC** GTG ACT GGA GTT CAG ACG TGT GCT CTT CCG ATC
RPI12	CAA GCA GAA GAC GGC ATA CGA GAT **TACAAG** GTG ACT GGA GTT CAG ACG TGT GCT CTT CCG ATC
RPI13	CAA GCA GAA GAC GGC ATA CGA GAT **TTGACT** GTG ACT GGA GTT CAG ACG TGT GCT CTT CCG ATC
RPI14	CAA GCA GAA GAC GGC ATA CGA GAT **GGAACT** GTG ACT GGA GTT CAG ACG TGT GCT CTT CCG ATC
RPI15	CAA GCA GAA GAC GGC ATA CGA GAT **TGACAT** GTG ACT GGA GTT CAG ACG TGT GCT CTT CCG ATC
RPI16	CAA GCA GAA GAC GGC ATA CGA GAT **GGACGG** GTG ACT GGA GTT CAG ACG TGT GCT CTT CCG ATC

Reverse adapter primers with unique index sequences shown in **red**
